# Macrophages Phenotype Regulated by IL-6 Are Associated with the Prognosis of Platinum-Resistant Serous Ovarian Cancer: Integrated Analysis of Clinical Trial and Omics

**DOI:** 10.1155/2023/6455704

**Published:** 2023-04-19

**Authors:** Xiaoqing Wu, Wenping Lu, Chaojie Xu, Cuihong Jiang, Zhili Zhuo, Ruipeng Wang, Dongni Zhang, Yongjia Cui, Lei Chang, Xi Zuo, Ya'nan Wang, Heting Mei, Weixuan Zhang, Mengfan Zhang, Chen Li

**Affiliations:** ^1^Department of Oncology, Guang'anmen Hospital, China Academy of Chinese Medical Sciences, Beijing 100053, China; ^2^The Fifth Affiliated Hospital of Zhengzhou University, Zhengzhou University, Zhengzhou 450052, Henan Province, China; ^3^Department of Oncology, Guang'anmen Hospital South Campus, China Academy of Chinese Medical Sciences, Beijing 102627, China; ^4^Department of Biology, Chemistry, and Pharmacy, Free University of Berlin, Berlin 14195, Germany

## Abstract

**Background:**

The treatment of platinum-resistant recurrent ovarian cancer (PROC) is a clinical challenge and a hot topic. Tumor microenvironment (TME) as a key factor promoting ovarian cancer progression. Macrophage is a component of TME, and it has been reported that macrophage phenotype is related to the development of PROC. However, the mechanism underlying macrophage polarization and whether macrophage phenotype can be used as a prognostic indicator of PROC remains unclear.

**Methods:**

We used ESTIMATE to calculate the number of immune and stromal components in high-grade serous ovarian cancer (HGSOC) cases from The Cancer Genome Atlas database. The differential expression genes (DEGs) were analyzed via protein–protein interaction network, Kyoto Encyclopedia of Genes and Genomes (KEGG) and gene ontology (GO) analysis to reveal major pathways of DEGs. CD80 was selected for survival analysis. IL-6 was selected for gene set enrichment analysis (GSEA). A subsequent cohort study was performed to confirm the correlation of IL-6 expression with macrophage phenotype in peripheral blood and to explore the clinical utility of macrophage phenotype for the prognosis of PROC patients.

**Results:**

A total of 993 intersecting genes were identified as candidates for further survival analysis. Further analysis revealed that CD80 expression was positively correlated with the survival of HGSOC patients. The results of GO and KEGG analysis suggested that macrophage polarization could be regulated via chemokine pathway and cytokine–cytokine receptor interaction. GSEA showed that the genes were mainly enriched in IL-6-STAT-3. Correlation analysis for the proportion of tumor infiltration macrophages revealed that M2 was correlated with IL-6. The results of a cohort study demonstrated that the regulation of macrophage phenotype by IL-6 is bidirectional. The high M1% was a protective factor for progression-free survival.

**Conclusion:**

Thus, the macrophage phenotype is a prognostic indicator in PROC patients, possibly via a hyperactive IL-6-related pathway, providing an additional clue for the therapeutic intervention of PROC.

## 1. Introduction

Epithelial ovarian cancer (EOC) is a gynecological malignant tumor with the highest mortality rate. The most common pathological type is high-grade serous ovarian cancer (HGSOC). At the time of diagnosis, most patients have advanced-stage disease. Despite debulking surgery and platinum-based chemotherapy, the majority of the patients will experience disease recurrence requiring further treatment. Platinum-based chemotherapy is currently the main treatment. However, about 30% of the patients experience platinum resistance during their first-line treatment. Patients with recurrent EOC are typically categorized as having either platinum-resistant or platinum-sensitive disease based on a platinum-free interval of less than or greater than 6 months. Even for patients sensitive to platinum drugs after their initial treatment, progression-free survival (PFS) will gradually shorten after multiline treatments and multiple relapses. Eventually, most patients will develop resistance to platinum and become incurable. Thus, prognostic indicators and biomarkers of treatment need to be excavated to prepare for the discovery of new drugs. Therefore, it is urgently needed to develop novel, effective prognostic indicators and therapeutic strategies against platinum-resistant ovarian cancer (PROC) to improve prognosis.

Cancer development and progression occur in concert with alterations in the surrounding stroma. The tumor microenvironment (TME) is complex and constantly evolving. In addition to stromal cells, fibroblasts, and endothelial cells, the TME comprises innate and adaptive immune cells. These innate immune cell types include macrophages, dendritic cells, neutrophils, myeloid-derived suppressor cells, natural killer cells, and innate lymphoid cells. Moreover, a plethora of cytokines within the TME regulates immune functions that culminate in muted immune responses that guide tumor progression [1].

Macrophages are key components of the TME characterized by their variable phenotypes and diverse functions [2]. Macrophages modulate immune responses through pathogen phagocytosis and antigen presentation and also function in wound healing and tissue repair, thus necessitating them for immune homeostasis [3]. High macrophage infiltration of most tumor types, including breast cancer, gastric cancer, lung cancer, hepatoma, and other malignancies, correlated with a negative prognosis, further establishing their role in cancer progression [4–6]. Ongoing clinical trials that target macrophages receptor, CSF-1R, and CCL2–CCR2 signaling axis show promising ablation of tumor-infiltrating macrophages in advanced solid tumors [7]. CSF-1R inhibitors can quickly deplete macrophages in tumors and reconstruct tumor microenvironments [8, 9]. Afterward, researchers found that not only the abundance of macrophages can affect the prognosis, but also the phenotype of macrophages is one of the important prognostic indicators. At present, the classification of macrophage phenotype is two extreme forms of macrophages in vitro, called by M1 and M2. Although the classification has been controversial recently, it is still meaningful and irreplaceable to explore the impact of macrophage phenotype on prognosis, especially in cohort studies [10]. The role of macrophage phenotype changes in predicting the prognosis of HGSOC patients and the mechanism underlying macrophage polarization at TME has remained unclear. Therefore, bioinformatics analysis of differential expression of TME-related genes in HGSOC and normal ovarian tissue specimens, correlation of macrophage phenotype and prognostic outcome, and the mechanism of macrophage polarization was performed in our study. A cohort study, including PROC patients, was conducted to further explore the effect of macrophage phenotype on prognosis and to reveal the regulation of macrophage phenotype in PROC.

## 2. Materials and Methods

### 2.1. Raw Data

Transcriptome RNA-seq data of 374 HGSOC cases (all data are tumor samples) and the corresponding clinical data were downloaded from The Cancer Genome Atlas (TCGA) database (https://portal.gdc.cancer.gov/).

### 2.2. Calculation of ImmuneScore, StromalScore, and ESTIMATEScore

ESTIMATE algorithm by the feat of R language version 4.1.1 loaded with estimate package was used to estimate the ratio of immune-stromal component in TME for each sample, exhibited in the form of three kinds of scores: ImmuneScore, StromalScore, and ESTIMATEScore, which positively correlated with the ratio of immune, stromal, and the sum of both, respectively, which means the higher the respective score, the larger the ratio of the corresponding component in TME.

### 2.3. Generation of Differential Expression Genes (DEGs) between High-Score and Low-Score Groups regarding ImmuneScore and StromalScore

All tumor samples were labeled with high score or low score depending on the comparison to the median score in regards to ImmuneScore and StromalScore, respectively. Package limma was used to perform differentiation analysis of the gene expression, and DEGs were generated by the comparison between the high-score samples vs. the low-score samples. DEGs with fold change larger than 1 after transformation of logFC (high-score group/low-score group) and false discovery rate (FDR) < 0.05 were considered significant [11]. Heatmaps of DEGs were produced by R language with package pheatmap. LogFC ≥ 1 represents the gene upregulation in tumor tissue, logFC ≤ −1 represents gene downregulation.

### 2.4. Gene Ontology (GO) and the Kyoto Encyclopedia of Genes and Genomes (KEGG) Enrichment Analyses

GO and KEGG enrichment analyses using 374 DEGs were performed with R language with the aid of packages clusterProfiler, enrichplot, and ggplot2. Terms with FDR-value of <0.05 were considered significantly enriched.

### 2.5. Construction of Protein–Protein Interaction (PPI) Network

PPI network was constructed by STRING database, followed by reconstruction with Cytoscape of version 3.6.1. Nodes with the confidence of interactive relationship larger than 0.95 were used for building network. The number of adjacent nodes of each gene was counted by R.

### 2.6. Kaplan–Meier Survival Analysis of HGSOC Patients

We conducted survival analysis by using R package “survminer.” A total of 374 samples were included in the survival analysis, and five samples were excluded due to lack of clinical data. The samples were divided into high-expression group and low-expression group according to the median value of CD80 gene expression. The survival curve was drawn by Kaplan–Meier method. Log-rank was used to test the statistical significance between the two groups, *P* < 0.05 was considered to be significant.

### 2.7. Gene Set Enrichment Analysis

KEGG and Hallmark were downloaded from Molecular Signatures Database as the target sets with which gene set enrichment analysis (GSEA) was performed using the software GSEA-4.1 downloaded from Broad Institute [12]. The whole transcriptome of all tumor samples was used for GSEA, and only gene sets with FDR *q* < 0.05 were considered significant.

### 2.8. Correlation between Tumor-Infiltrating Macrophages and IL-6

Gepia database (http://gepia.cancer-pku.cn/index.html) was applied for estimating the macrophages abundance in ovarian cancer tissues, which was followed by purity adjustment and Spearman's correlation. *P* < 0.05 was considered a statistical difference. *R* > 0 was considered a positive correlation, and *R* < 0 was considered a negative correlation between the two subjects.

### 2.9. Cohort Study to Explore the Relationship between Macrophage Phenotype and Prognosis in PROC

A total of 75 patients with PROC were included in the prospective cohort study. The cases come from patients who were treated the outpatients and inpatients in the oncology department of Guang'anmen Hospital, China Academy of Chinese Medical Sciences, from May 2021 to December 2021. The end point of observation was the disease progression or death of the patient. For patients with measurable solid tumors, the identification of disease progression is based on RECIST 1.1. For patients without measurable solid tumors, CA125 exceeded twice the upper limit of normal value and lasted for more than 1 week, or ovarian cancer cells are found in ascites, we identified that the disease is progressing. The study has been registered the ethical review of the ethics committee of Guang'anmen Hospital, ethical review No.: 2021-019-ky. This study has been registered in China clinical trial registration center with registration number: ChiCTR2100051316.

Diagnostic criteria: According to the patient's medical history, symptoms and signs, imaging evidence (B-ultrasound, pelvic and abdominal computed tomography, nuclear magnetic resonance, etc.), pathological results (ascites cytology, tumor histological examination, etc.), and tumor marker examination, it is determined that the patient is one with serous ovarian cancer.

Inclusion criteria: (1) the patient was diagnosed as primary epithelial HGSOC by pathological examination; (2) it recurred within 6 months after the last platinum-based chemotherapy or progressed during the chemotherapy, which was clinically determined to be platinum resistant or platinum refractory; (3) ECoG score: 0–2; (4) estimated survival time ≥3 months; (5) sign informed consent.

Exclusion criteria: (1) previous history of other tumors; (2) patients receiving antitumor therapy; (3) patients with infectious diseases or immune system diseases have not yet been controlled; (4) recent use of immunosuppressants; (5) suffering from serious heart, liver, kidney, and hematopoietic diseases without control; (6) those who cannot cooperate with follow-up, such as severe neurological deficit (such as aphasia and agnosia), mental illness, or other reasons.

### 2.10. Blood Draw, Measurement of IL-6, and Flow Cytometry

At the time of enrollment, 10 mL of fasting venous blood was collected by nurses, and the laboratory department of Guang'anmen Hospital was entrusted to detect IL-6, the percentage of M2 and M1 in peripheral blood. M1 was labeled with CD80, M2 was labeled with CD206, and the proportion of M2 and M1 in human peripheral blood monocyte-macrophage subsets were detected by flow cytometry. We performed a scatter diagram with IL-6 as the abscissa and M2 ratio as the ordinate and drew the fitting line. Then we set the abscissa of the symmetry axis of the parabola as the cutoff value of IL-6.

### 2.11. Immunohistochemistry in Tissues

Immunohistochemistry of core protein was utilized to confirm the expression in normal ovary and ovarian cancer tissue in The Human Protein Atlas (https://www.proteinatlas.org).

### 2.12. Statistical Analysis

SPSS 25.0 was used for analyses, and descriptive statistics were used to analyze all data, including demographic data, baseline, various efficacy evaluation indicators, etc. Describe the mean and standard deviation of measurement data, and calculate the 95% confidence interval of the mean. For count data, describe the frequency and percentage, and calculate the 95% confidence interval for the percentage. The measurement data were compared within the group according to whether they conformed to normality, using paired data paired *t*-test or Wilcoxon rank-sum test. Wilcoxon rank-sum test was used for grade data analysis, and *t*-test was used for count data comparison. Kaplan–Meier method was used for PFS analysis, and log rank test was used to compare the PFS of the two groups.

## 3. Results

### 3.1. Identification of DEGs Based on ImmuneScore and StromalScore

**Figure 1:**
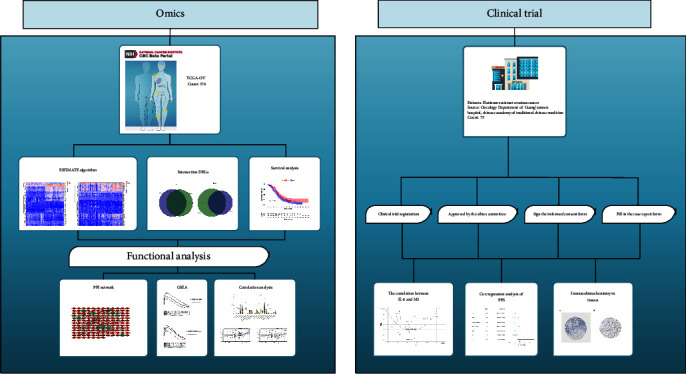
Graphical abstract of integrated analysis of clinical trial and omics.

**Figure 2:**
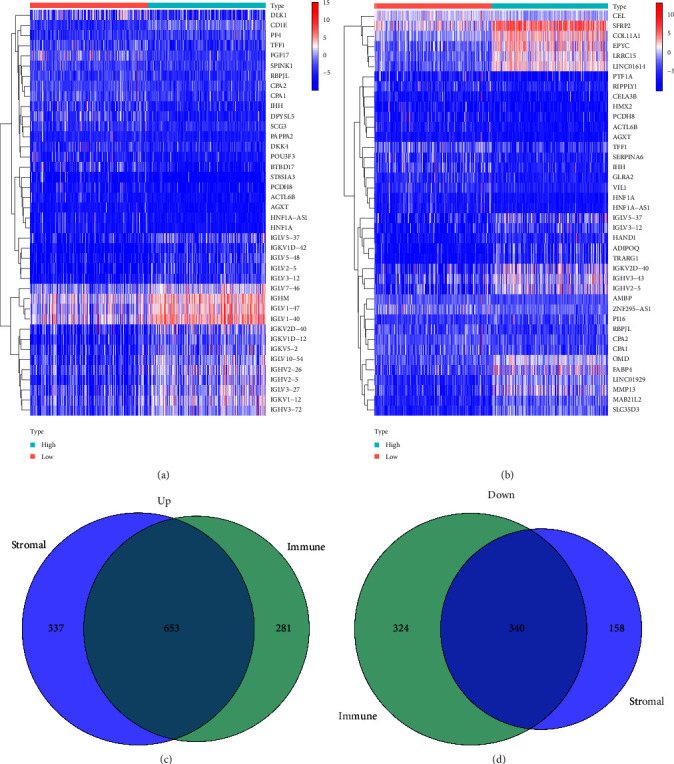
Heatmap (blue: downregulation; red: upregulation) for DEGs. (a) Heatmap generated by comparison of the high-score group vs. the low-score group in ImmuneScore. Row name of heatmap is the gene name. (b) Heatmap generated by comparison of the high-score group vs. the low-score group in StromalScore. (c) and (d) Venn plots showing common upregulated and downregulated DEGs shared by ImmuneScore and StromalScore.

Graphical abstract of the study is shown in Figure 1. We extracted 374 cases in TCGA database; all of them were samples of HGSOC tissue. To ascertain the exact alterations of gene profiles in TME regarding immune and stromal components, we conducted a comparative analysis between high- and low-score samples. Compared to the median, a total of 1,598 and 1,488 DEGs were identified from ImmuneScore and StromalScore, respectively (samples with high score vs. low score). Heatmaps were also generated for DEGs from ImmuneScore and StromalScore (Figures 2(a) and 2(b)).

**Figure 3:**
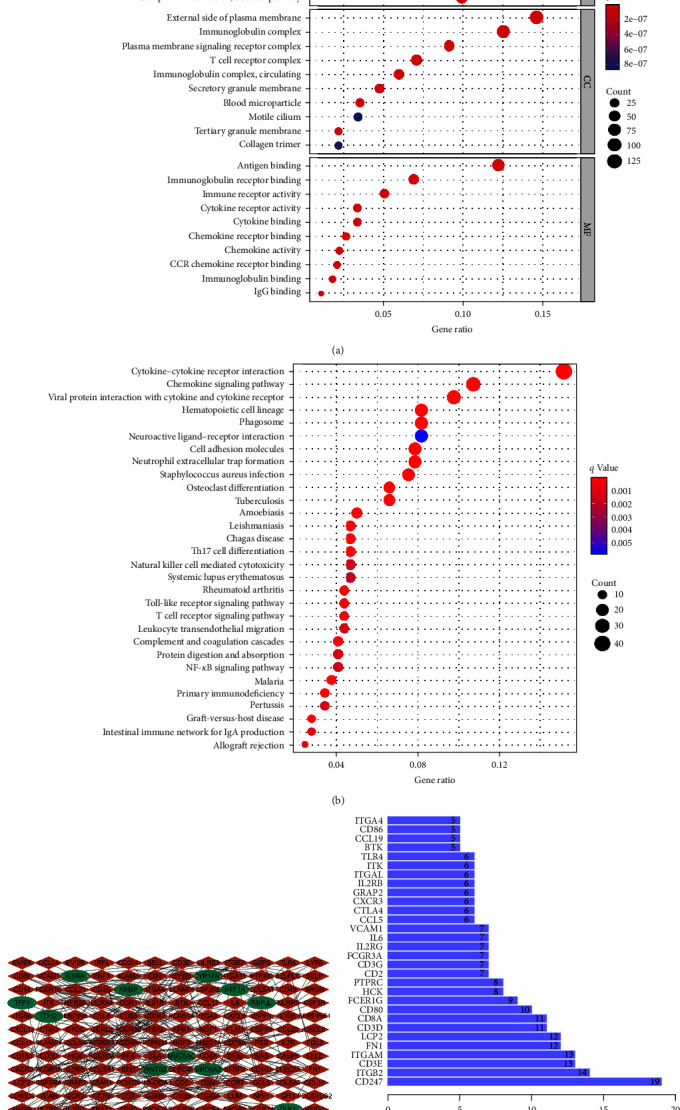
Functional enrichment analyses of DEGs. (a) GO enrichment analysis. (b) KEGG enrichment analysis. (c) Protein–protein interaction network. Brown: upregulation; Green: downregulation. (d) The top 30 genes ordered by the degree of nodes in PPI network.

The intersection of ImmuneScore and StromalScore contained 653 upregulated and 340 downregulated DEGs (Figures 2(c) and 2(d)). Subsequent GO enrichment analysis indicated that almost all 993 DEGs were mapped to the immune-related GO terms, such as adaptive immune response based on somatic recombination of immune receptors built from immunoglobulin superfamily domains, immune response-activating cell surface receptor signaling pathway, immune response-activating signal transduction, and lymphocyte-mediated immunity (Figure 3(a)). KEGG analysis identified a wide spectrum of significantly enriched genes involved in chemokine signaling pathway, cytokine–cytokine receptor interaction, hematopoietic cell lineage, and viral protein interaction with cytokine and cytokine receptors (Figure 3(b)). Thus, functions of these DEGs seemed to relate to cytokine-regulated immune response and immunocyte production, implying that the enrichment of immune factors was a predominant feature of TME in HGSOC.

### 3.2. Increased CD80 and IL-6 Expression in TME of HGSOC

Among 653 upregulated DEGs, IL-6 and CD80 were particularly enriched in pathways involved in adaptive immune response and lymphocyte-mediated immunity. Notably, increased expression of these two genes was also identified by KEGG analysis. We further conducted the PPI network analysis based on the STRING database. The interaction genes obtained from PPI network analysis were intersected with 993 common DEGs to obtain a final panel of 151 genes. The logFC of CD80 and IL-6 was 1.433 and 1.233, respectively, suggesting that CD80 and IL-6 were upregulated in HGSOC tumor issues. The interactions between 151 genes are shown in Figure 3(c) using Cytoscape software, and the bar plots were represented for the top 30 genes ranked by degree (the number of adjacent nodes), as shown in Figure 3(d). In the interaction network, we mainly pay attention to those nodes of high degree and high betweenness. The values for degree and betweenness of CD80 are 10 and 0.1055, ranking 9th and 17th, respectively, among 151 genes. The values for degree and betweenness of IL-6 are 7 and 0.2009, ranking 13th and 9th, respectively. Put together, the aforementioned data clearly reveals that IL-6 and CD80 are upregulated at both mRNA and protein expression levels, indicating that they may play critical roles in TME formation, regulation, and maintenance.

### 3.3. Correlation of CD80 and IL-6 Expression with HGSOC Patients' Survival and TME Modulation

**Figure 4:**
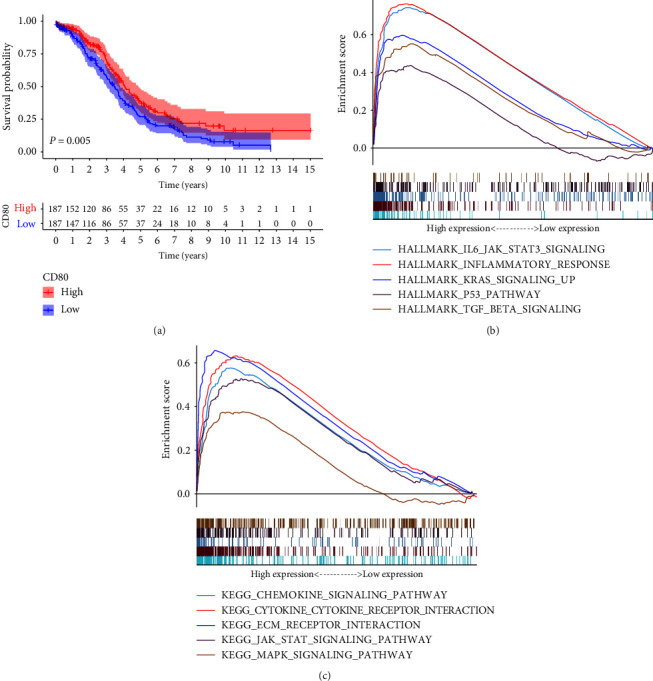
Survival analysis and GSEA. (a) Survival analysis for HGSOC patients with different CD80 expression. Patients were labeled with high expression or low expression depending on the comparison with the median expression level. (b) GSEA for samples with high IL-6 expression and low expression. Each line representing one particular gene set with unique color, and upregulated genes located in the left, approaching the origin of the coordinates; by contrast, the downregulated lay on the right of *x*-axis. Only gene sets with NOM *P* < 0.05 and FDR *q* < 0.06 were considered significant. (c) The enriched gene sets in HALLMARK collection by the high IL-6 expression sample. (d) Enriched gene sets in KEGG by samples of low IL-6 expression.

In the present study, we conducted a survival analysis of all HGSOC patients classified into CD80 high-expression group and low-expression group compared with the CD80 median expression. The previous results indicated that the expression of CD80 in TME was positively correlated with the prognosis of HGSOC patients (Figure 4(a)). Given CD80 is a surface marker of M1; we deduced that subtypes of macrophage mediated by IL-6 in TME can affect the prognosis of HGSOC patients.

Similarly, GSEA analysis of IL-6 was implemented in the high-expression and the low-expression groups compared with the median level of IL-6 expression at 0.524. The genes in IL-6 high-expression group were mainly enriched in immune-related activities, such as inflammatory response and IL-6-JAK-STAT3 signaling pathway, and typical tumor pathways, such as KRAS, P53, and TGF-*β* pathway (Figure 4(b)). The enrichment score of IL-6-JAK-STAT3 signaling pathway is 0.745, compared with IL-6 low-expression group, *P* = 0.001. As to IL-6 low-expression group, the aforementioned genes were not significantly enriched.

In KEGG analysis, the genes were enriched in chemokine signaling pathway, cytokine–cytokine receptor signaling, JAK-STAT, and MAPK pathways (Figure 4(c)). The enrichment score of JAK-STAT pathway is 0.546, compared with IL-6 low-expression group, *P* = 0.003. This indicates that the activation of this pathway is positively correlated with the expression of IL-6. IL-6-JAK-STAT3 pathway is closely related to the composition and changes of TME based on previous studies [13–15].

**Figure 5:**
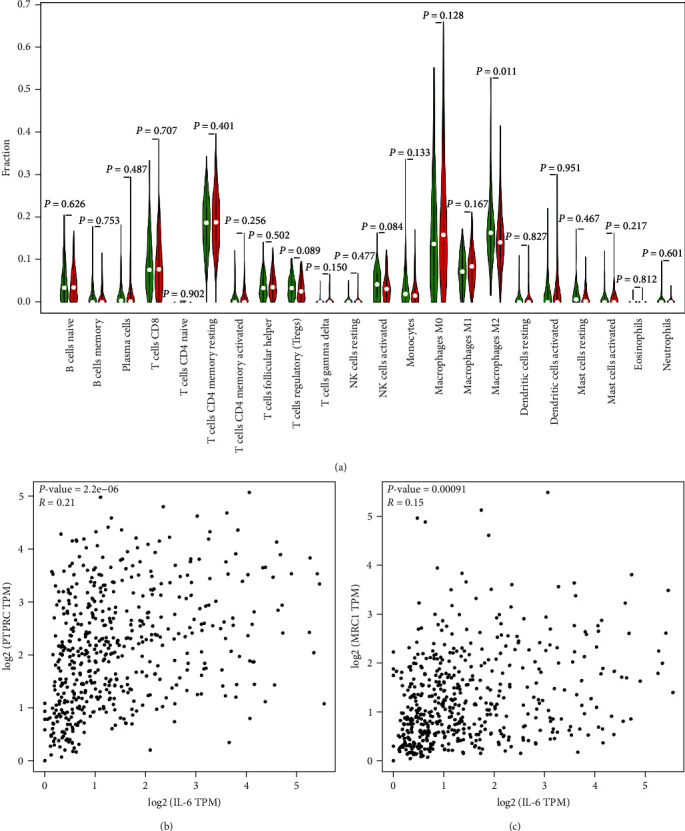
The correlation between IL-6 and macrophage. (a) Difference in distribution of immune cells between high- and low- expression groups of IL-6. Red: high-expression group; Green: low-expression group. (b) and (c) The positive correlation between IL-6 and macrophages and M2. CD45/PTPRC is widely recognized as a surface marker for macrophages, and CD206/MRC1 is widely recognized as a surface marker for M2.

We further confirm IL-6 expression with the immune cells, the proportion of tumor-infiltrating immune subsets was analyzed using CIBERSORT algorithm, and 22 kinds of immune cell profiles in EOC samples were constructed (Figure 5(a)). The results from the difference and correlation analyses showed that M2 macrophages were correlated with the expression of IL-6. The proportion of tumor-infiltrating macrophage subsets was analyzed using Timer 2.0 database. The results from the correlation analyses showed that macrophages and M2 macrophages were positively correlated with the expression of IL-6 (Figures 5(b) and 5(c)). With the increase of IL-6, the abundance of tumor-infiltrated monocytes/macrophages and M2 macrophages increased after purity adjustment (monocytes/macrophages: *P* < 0.001, M2 macrophages: *P* < 0.001). These data suggested that IL-6 pathway might be a potential indicator for the status of TME.

### 3.4. Determination of IL-6 Level and Macrophages Abundance in Peripheral Blood

A total of 75 HGSOC patients participated in a cohort study to have their IL-6, M1, and M2 macrophages in peripheral blood measured. The baseline situation of these patients is shown in Table 1. As shown in Supplementary Figure S1, IL-6 levels of all included patients and the corresponding proportions of M2 macrophages (M2%) are drawn in the form of a scatter plot. Each point can fit into a parabolic curve after nonlinear logistic regression analysis, and the arranging center axis absorbent is 5.5 ng/L, i.e., the cutoff value by which the HGSOC patients were categorized into two groups. There were 52 patients with IL-6 equal or below 5.5 ng/L and 23 patients with IL-6 above 5.5 ng/L, respectively. The data analysis showed that when the IL-6 level was below the concentration, the proportion of M2 and M2/M1 ratio decreased as IL-6 increased (Pearson coefficient < 0). When IL-6 level was above the concentration, the proportion of M2 and M2/M1 ratio increased as IL-6 increased (Pearson coefficient > 0). Although there was no correlation between the expression of IL-6 and the percentage of M1 (M1%), *P* > 0.05, we seem to be able to observe a few trends. When the IL-6 level was below the concentration, Pearson coefficient between IL-6 and M1% are greater than 0. When IL-6 level was above the concentration, Pearson coefficient between IL-6 and M1% is less than 0 (Table 2).

### 3.5. Lines of Therapy and M1 Abundance are Related to PFS

**Figure 6:**
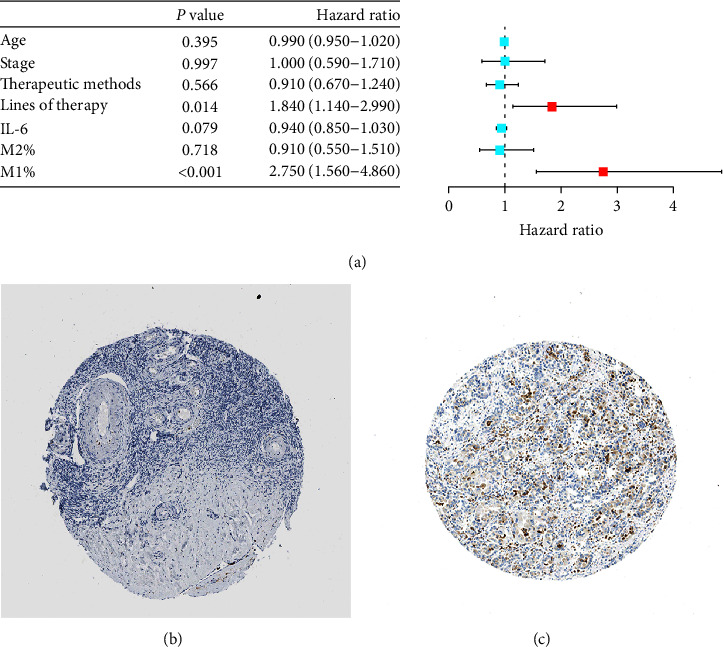
The influence factor of PFS and comparison of M2 infiltration in tissues. (a) The forest plot of influence factor of PFS by Cox regression. (b) The infiltration of M2 in ovarian tissue. (c) The infiltration of M2 in ovarian cancer tissue.

With patients' baseline situation shown in Table 1, Cox regression analysis was conducted on the age, clinical stage, therapeutic methods, lines of therapy, IL-6, and the proportion of M1 and M2 to obtain the influencing factors that may be related to their PFS. The age and the percentage of M2 conform to the normal distribution. Subsequently, we evaluated the impact of these factors on PFS one-by-one using multivariate Cox regression analysis, and the results are shown in Table 3, Figure 6(a). The lines of therapy and M1% were found to correlate with PFS. The line of therapy was a risk factor of PFS in PROC patients (hazard ratio (HR): 2.64, 95% confidence interval (CI): 1.04–6.64, *P* < 0.05). In contrast, M1% was a protective factor of PFS (HR: 2.75, 95% CI: 1.56–4.86, *P* < 0.001). M2%, clinical stage (Ⅲc, Ⅳ), age, and methods (chemotherapy, targeted, and targeted combined chemotherapy) were not related to PFS (*P* > 0.05).

### 3.6. Differential Expression of M2 in Normal Tissues and Ovarian Cancer Tissues

In accordance with the TPA database, Figures 6(b) and 6(c) illustrate the expression of M2. The expression of M2 was more expressive in ovarian tumorous tissue as compared to healthy ovarian tissues.

## 4. Discussion

In summary, we attempted to identify TME-related genes contributing to the survival of HGSOC patients from the TCGA database. We found that these differentially expressed genes exist mainly in immune response pathways. In particular, CD80 expression could be potentially used to predict the prognosis of EOC, indicating that survival maybe related to phenotype changes of macrophages in TME via possible activation of IL-6-STAT3 pathway. Furthermore, additional data from our following clinical study found that the regulatory effect of IL-6 on M2 macrophages can be either stimulatory or suppressive depending on its level in the circulating system of patients with PROC. Since TME can be changed from tumor-friendly to tumor-suppressive by switching phenotypes from M2 to M1, IL-6-mediated phenotypic transformation of macrophages may serve as a novel therapeutic target in the PROC.

Dynamic presence of macrophages of variable phenotypes contributes to the remodeling of TME. Macrophages can be divided into two different subgroups according to different inducible factors, classical or inflammatory M1 macrophages and alternative or anti-inflammatory M2 macrophages. M1 macrophages expressing surface marker CD80 can secrete a number of pro-inflammatory factors. M2 macrophages are marked with CD206 and can be classified into four subtypes: M2a, M2b, M2c, and M2d. They can secrete anti-inflammatory factors, tissue repair factors, and angiogenesis factors [10, 16, 17]. The latest research found that the previous classification method was characterized by two extreme states of macrophages, which usually correspond to in vitro conditions. In vivo, macrophages may be in an intermediate state. But the explanation for the link between TAM definition and function still relies heavily on the M1–M2 paradigm, and the classification was widely known and agreed. There are two sources of tumor-associated macrophages in TME: embryonic and adult hematopoiesis-derived tissue-resident macrophages and monocyte circled-derived macrophages. At the molecular level, recruited macrophages predominate in the TME in advanced tumors [18]. The study explained why we also found the polarization of macrophages in peripheral blood. The contributions of M1-like macrophages to tumor development are double-edged. At the early stage of tumor initiation, TAMs are M1-like phenotypes before transferring to the M2-like type. M1 can play an antitumor role by secreting inflammatory factors to activate immunity. While it also enhances the metastatic potential of cancer cells via activating NF-*κ*B signals [19]. M2-type can especially promote angiogenesis which facilitates tumor growth and metastasis due to the favorable nutrient and oxygen transportation, and tissue remolding and immunosuppression as well. Immune-suppressive M2 macrophages are alternatively activated by cytokines such as IL-6. In the early stage, the local infiltrating macrophages are predominantly M1, while in the late stage of the tumor, the macrophages infiltrating around the tumor are mostly M2 type [20, 21]. Previous studies have shown that IL-6 activates both M1 and M2 [22–24]; however, the difference in macrophage phenotypes associated with clinical staging suggests that there are other factors, including the amount of IL-6, that influence the direction of polarization of macrophages. The results of our study showed that IL-6 reduces the percentage of M2 and M2/M1 below a certain level and increases the percentage of M2 and M2/M1 ratio above a certain level in patients with advanced PROC, a higher abundance of M1 predicted a better prognosis. This phenomenon suggests that the direction of IL-6 regulation of macrophage polarization is very complex and is related to the amount of IL-6. M1 and M2 are not necessarily mutually exclusive in advanced ovarian cancer but coexist, producing a mixed phenotype that tends to be functional depending on the balance of activating and inhibiting activities and the tumor environment [25]. In advanced patients, the macrophages infiltrating around the tumor are mostly M2 type, the immunosuppressive state is serious in TME, and M1 plays a role in activating immunity. In other words, it may be meaningful and beneficial to inhibit the transformation of macrophages from M1 to M2 in advanced patients.

In recent years, researchers have found that the different phenotypic expressions of macrophages in the microenvironment will actually lead to discrepant prognoses. Corvigno et al. [26] found that high CD80 density in 119 cases of HGSOC was significantly correlated with longer PFS. Hwang et al. [27] evaluated the ratio of M1 and M2 in 349 lung cancer tissues and found that elevated levels of M1 macrophages at the primary tumor sites indicated a better prognosis [28], while increasing levels of M2 are associated with poor survival [29, 30]. Compared with benign ovarian neoplasms, macrophages in malignant tissues are inclined to transform from M1 into M2 [31]. These results again showed that patients with high infiltration of M1 into tumor stromal tissues had a better prognosis, and a high ratio of M2 was a powerful indicator of poor prognosis. In observing 140 patients with advanced high-grade plasmacytic ovarian cancer, as well as patients with other histotypes of ovarian cancer and ovarian metastases from other sites, Antonio Macciò found that higher M1/M2 ratios were associated with longer platinum-free intervals, overall survival, and PFS [32]. Some studies have also found that tumor-associated macrophage-related molecular markers and secreted cytokines, such as CD163, CD206/CD68, and IL-10, were related to the prognosis of HGSOC [30]. In Kaplan–Meier analysis of TCGA data, we found that CD80 is related to the prognosis of HGSOC. This finding makes sense as CD80 is a specific surface marker of M1 macrophage. The correlation of a higher level of CD80 expression with longer survival was further confirmed in our clinical trial as the proportion of M1 was found to correlate with PFS in PROC patients, generally in agreement with previous reports [26–28]. Thus, these results suggest that the relative abundance of macrophage phenotypes may serve as an important prognostic factor for PROC.

Patients with recurrent ovarian cancer are typically categorized as platinum-resistant or platinum-sensitive, based on a platinum-free interval of less than or greater than 6 months. NCCN guideline [33] suggested that the PROC is mainly treated by nonplatinum chemotherapy and targeted therapy. Patients with PROC have low response rates to subsequent chemotherapy (<15%), with a PFS of 3–4 months and a median survival of under a year [34]. Most of the response rates of targeted therapies from patients with PROC were also very low at <10% [35], except for that of bevacizumab at 27.3% [36]. Tumor-associated macrophages play an important role in platinum resistance [37]. Tumor-associated macrophages, most of them are M2 [38], may produce ascitic fluid lysophosphatidic acid (LPA) in EOC, and LPA can promote survival, proliferation, migration, and platinum resistance [39]. M0- and M1-type macrophages showed significant transcriptional alterations in the transformation of platinum-sensitive into PROC cell lines [40]. IL-6 is reportedly secreted by tumor cells and cancer-associated fibroblasts in response to platinum treatment, enhancing chemoresistance via STAT3-mediated enrichment of cancer stem cells [41]. Hence, IL-6 is an important cytokine and chemokine closely related to the development of platinum resistance in EOC [42, 43]. Our study demonstrates that such an effect of platinum resistance can be further enhanced when IL-6 level is further increased above a threshold value of 5.5 ng/L in the peripheral blood of PROC patients. We hypothesize that a higher concentration of IL-6 encourages M1 to M2 transformation of macrophages in TME, increases the recruitment of macrophages at tumor sites, and further activates the feedback loop between IL-6 and M2 to promote tumor growth and survival [44]. We propose that by blocking IL-6 activity at TME, this transformation will be reversed, and the proportion of M1 will increase to hence prohibit tumor growth and development. Thus, macrophage phenotype transformation is an attractive target or as an alternative therapeutic strategy for PROC.

### 4.1. Lysophosphatidic Acid

In our study, we did not find a prognostic effect of IL-6. We are aware of some literature reporting IL-6 as an independent risk factor for prognosis in cancer patients [45–47]. There are two possible reasons for this inconsistency: one is that our population was late progressive patients, unlike those included in the literature who had early stable disease, and the second is that our sample size was relatively small. Although IL-6 had no effect on prognosis, we also found a relationship between IL-6 and macrophage phenotype. We further analyzed the mechanism via which IL-6 could trigger macrophage polarization at TME. The GSEA results showed that pathways of IL-6-STAT3, JAK/STAT3, Ras-MAPK, and some inflammation factors were significantly enriched in the IL-6 high-expression group. Many researchers have found that IL-6-related pathways are involved in the regulation of macrophage phenotypes. Yin et al. [48] found that IL-6/STAT3 pathway was inhibited in M1 macrophages, while IL-6/STAT3 pathway was activated in M2 macrophages. In macrophages treated with IL-6-specific antibodies, they found that the macrophages transformed into M1-type as a result of inhibition of IL-6/STAT3 pathway. Furthermore, when human macrophages were cocultured with IL-6, they became polarized toward M2-like macrophages [49]. Weng et al. [50] and Zhang et al. [51] found that oncogene *MCT-1* could stimulate IL-6 expression that, in turn, promoted THP-1 monocytes to polarize into M2-like macrophages. The mechanism maybe due to the hyperactive IL-6-STAT3 signaling pathway. However, some studies have also shown the positive effect of IL-6 on the immune system. M1 secretes IL-6 for pro-inflammatory response [23, 52]. IL-6 promotes anti-inflammatory responses by inducing the expression of IL-4, resulting in the differentiation of recruited T cells into Th2 [53]. Th2 cells can promote the proliferation and differentiation of B cells and the production of antibodies. IL-6 pathway was found to induce the expression of T-cell chemokines such as CCL4, CCL5, CCL17, and CXCL10 [8]. In addition, IL-6 has been proved to have an impact on the activation, expansion, survival, and polarization of T cells [9]. IL-6 prevents T-cell apoptosis by upregulating antiapoptotic factors (such as Bcl-2 and Bcl XL) and regulating the surface expression of Fas receptor depending on STAT3 [54, 55]. IL-6 can promote T-cell antigen receptors and stimulate T-cell proliferation [56]. In our clinical trial, we found that IL-6 could have two opposite correlations with the proportion of M2 at different concentrations. We found that IL-6 could promote M2-type transformation only when it was above a certain threshold value at 5.5 ng/L, indicating that excessive secretion and relatively high IL-6 in vivo maybe able to drive the differentiation of macrophages into M2-type. When IL-6 was below this concentration, it was inversely correlated with the abundance of M2 macrophages, suggesting that IL-6 may also have a positive antitumor effect against PROC.

Many studies have connected macrophage phenotypes in TME with the prognosis and recurrence of HGSOC [26–32]. However, it is difficult for clinicians to obtain platinum-resistant OC tissue in clinical practice. In the current study, we focused our attention on the influence of macrophage phenotype on prognosis in peripheral blood. We reached similar results with previous studies in TME [27–30]. The result corroborated with prior investigations that M1 was a protective factor in PFS, suggesting blood specimens can also be used to predict prognosis. Our results further confirmed the effect of macrophage phenotype on prognosis. Regulating macrophage phenotype may be a new target for the treatment of PROC. The finding may help to guide the choice of therapeutic drugs. For patients with a lower level of M1 macrophages in peripheral blood, we may take a more active approach to treat patients and encourage them to participate in clinical trials of targeted therapies that will increase M1 abundance. In addition, our results also provide clues for the discovery of new therapeutic drugs against HGSOC in the future.

Unlike previous studies, which have conducted experiments with THP-1 cells [43, 48, 49, 51] to explore the way that macrophage phenotype is regulated, we measured IL-6 and analyzed macrophage phenotypes in peripheral blood in PROC patients. Measurement of biomarkers in peripheral blood to reflect the status of TME may be more feasible in daily clinical practice.

Certainly, the current study has been conducted with certain limitations. In our investigation, the cutoff value of 5.5 ng/L was only derived from a small set of HGSOC cases. Moreover, since the number of macrophages in peripheral blood is limited, our flow cytometry analysis may contain nonnegligible errors. The clinical trial results only showed that there is a connection between macrophage phenotype and prognosis, further validation in a large-scale independent cohort is certainly warranted. The sample of omics is not the same as the clinical sample because the enrolled patients were not recommended for surgery; our study failed to analyze the phenotype of macrophages in ovarian cancer tissue, which led to the data on TME from the omics population cannot be extended automatically to the cohort sample.

Finally, the results of the current study demonstrated that CD80, the surface marker of M1, can regulate the tumor microenvironment and potentially be a prognostic indicator. The mechanism underlining such correlation between macrophage phenotype switching and HGSOC survival may be related to the activation of IL-6-STAT3 pathway and change of TME status. In the future, we will carry out in vivo and in vitro experiments to detect IL-6, STAT3, and their upstream and downstream targets to further elucidate the role of this particular pathway in modulating macrophage phenotypes in TME of HGSOC.

## Figures and Tables

**Table 1:** The baseline of patients in cohort study.

Characteristic	All patients (cases (%))
Total	75
Age	
≤58	38 (50.7)
>58	37 (49.3)
Stage	
IIIc	51 (68.0)
IV	24 (32.0)
Treatment	
Nonplatinum chemotherapy	42 (56.0)
Apatinib	16 (21.3)
Nonplatinum chemotherapy + Bevacizumab	17 (22.7)
Lines of therapy	
First	5 (6.7)
Second	6 (8.0)
Third and above	64 (85.3)

**Table 2:** Correlation analysis between IL-6 and macrophages.

Items		IL-6	M1	M2	M2/M1
IL-6 (IL-6 < 5.5 ng/L)	Pearson coefficient	1	0.04	−0.51	−0.39
	*P*	–	0.792	<0.001^*∗*^	0.005^*∗*^
	*n*	52	52	52	52

IL-6 (IL-6 > 5.5 ng/L)	Pearson coefficient	1	−0.30	0.64	0.70
	*P*	–	0.170	0.001^*∗*^	<0.001^*∗*^
	*n*	23	23	23	23

^*∗*^Statistically significant.

**Table 3:** Multivariate Cox regression analysis of influencing factors related to PFS.

Factors	HR (95% CI)	*P*
Age	0.99 (0.95–1.02)	0.395
Stage	1.00 (0.59–1.71)	0.997
Therapeutic methods	0.91 (0.67–1.24)	0.566
Lines of therapy	1.84 (1.14–2.99)	0.014^*∗*^
IL-6	0.94 (0.85–1.03)	0.079
M2%	0.91 (0.55–1.51)	0.718
M1%	2.75 (1.56–4.86)	<0.001^*∗*^
M2/M1	0.06 (0.00–1.12)	0.335

^*∗*^Statistically significant.

## Data Availability

The datasets (ANALYZED) for this study can be found in the TCGA database (https://portal.gdc.cancer.gov), TIMER 2.0 (http://timer.cistrome.org/), and The Human Protein Atlas (https://www.proteinatlas.org/).

## Supplementary Materials

Supplementary Materials Figure S1: Scatter plot of IL-6 and M2%.
