# Supplementary figures and images for: Macrophages Phenotype Regulated by IL-6 Are Associated with the Prognosis of Platinum-Resistant Serous Ovarian Cancer: Integrated Analysis of Clinical Trial and Omics
Source: J Immunol Res. 2023 Apr 19;2023:6455704. doi: 10.1155/2023/6455704 (PMC10132904; doi:10.1155/2023/6455704)

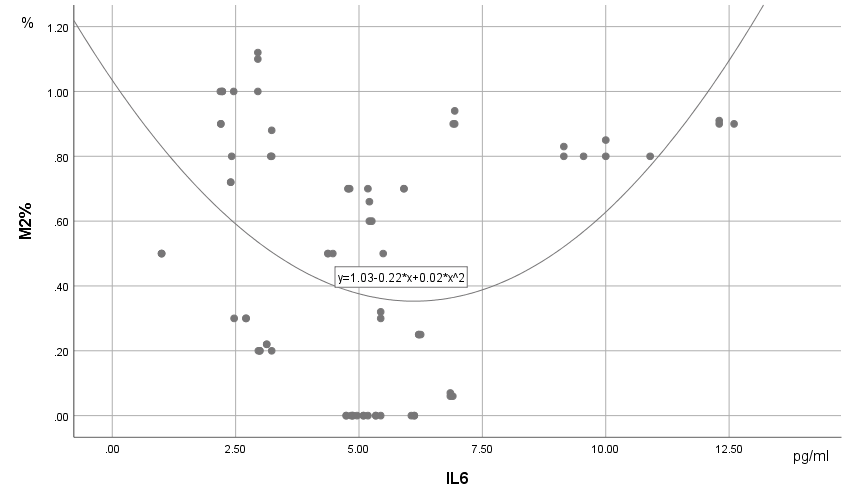

Supplement: Supplementary Materials — Figure S1: Scatter plot of IL-6 and M2%. [file 6455704.f1.png]
